# The Socio-Moral Image Database (SMID): A novel stimulus set for the study of social, moral and affective processes

**DOI:** 10.1371/journal.pone.0190954

**Published:** 2018-01-24

**Authors:** Damien L. Crone, Stefan Bode, Carsten Murawski, Simon M. Laham

**Affiliations:** 1 Melbourne School of Psychological Sciences, University of Melbourne, Melbourne, Australia; 2 Department of Finance, University of Melbourne, Melbourne, Australia; University of Vermont, UNITED STATES

## Abstract

A major obstacle for the design of rigorous, reproducible studies in moral psychology is the lack of suitable stimulus sets. Here, we present the Socio-Moral Image Database (SMID), the largest standardized moral stimulus set assembled to date, containing 2,941 freely available photographic images, representing a wide range of morally (and affectively) positive, negative and neutral content. The SMID was validated with over 820,525 individual judgments from 2,716 participants, with normative ratings currently available for all images on affective valence and arousal, moral wrongness, and relevance to each of the five moral values posited by Moral Foundations Theory. We present a thorough analysis of the SMID regarding (1) inter-rater consensus, (2) rating precision, and (3) breadth and variability of moral content. Additionally, we provide recommendations for use aimed at efficient study design and reproducibility, and outline planned extensions to the database. We anticipate that the SMID will serve as a useful resource for psychological, neuroscientific and computational (e.g., natural language processing or computer vision) investigations of social, moral and affective processes. The SMID images, along with associated normative data and additional resources are available at https://osf.io/2rqad/.

## Introduction

In fields such as affective science, large, diverse and systematically validated stimulus sets (e.g., [1–10]) have facilitated substantial scientific progress [11,12]. Such stimulus sets enable rigorous studies that can be easily compared, replicated and aggregated, and moreover, obviate the need for individual research groups to duplicate each other’s efforts in conducting labor-intensive stimulus validation (for discussion, see [13,14]). In the field of moral psychology, however, there is a clear shortage of standardized stimulus sets. This shortage places substantial constraints both on the kinds of paradigms that can feasibly be implemented, and on the reliability and validity of the conclusions researchers reach.

Motivated by this gap in the literature, we present the development and validation of a new picture set, the Socio-Moral Image Database (SMID). The SMID is the largest systematically validated moral stimulus set assembled to date, containing images with diverse moral content. This image set will facilitate a wide range of novel research, including basic inquiries into the cognitive and neural underpinnings of moral perception, evaluation and judgment [15–17], applied research on moral communication and persuasion [18–20], and large-scale computational investigations of moral content [21].

### Existing moral psychology stimulus sets

Currently, much moral psychology research relies on small, *ad hoc* stimulus sets, constructed with little (if any) prior validation. Such practices have several undesirable consequences. Specifically, *ad hoc* stimulus sets present obstacles to comparing and integrating findings across studies, and are relatively more vulnerable to unwanted confounds (e.g., see [22–25]). An obvious remedy is for moral psychology to employ standardized, well-validated materials, however only a small number of systematically validated moral stimulus sets exist (e.g., [26–29]; for a historically-oriented perspective, see [30,31]). Unfortunately, each suffers from important limitations.

Specifically, existing sets are often (1) brief, textual representations of moral content, (2) representative of only a narrow segment of the moral domain (e.g., sacrificial dilemmas) and normed on only a limited number of variables, (3) based on an assumption that each stimulus specifically represents one and only one class of moral content, or (4) restricted to one pole (typically the negative pole) of the moral spectrum. A summary of these features as they apply to prominent existing stimulus sets is presented in Table 1, along with a comparison to the SMID. In the following paragraphs, we elaborate on methodological challenges relating to each of these features, and how the SMID can contribute to overcoming them.

**Table 1:** Comparison of contemporary moral stimulus sets with the SMID.

Stimulus Set	Medium	*N* Stimuli	Breadth of Normative Data		Moral Valence		
			Moral Content	Non-Moral Content	Pos.	Neut.	Neg.
Chadwick et al. [27]^a^	Text	500	Each stimulus rated on *one and only one* of the following dimensions: Charitability, Uncharitability, Cooperativeness, Uncooperativeness, Honesty, Dishonesty, Loyalty, Disloyalty, Friendliness, Unfriendliness	−	×		×
Clifford et al. [26]^b^	Text	132	All stimuli rated on: Wrongness and *categorized as one of* a Care, Fairness, Ingroup, Authority, Purity, Liberty, or Other violation	All stimuli rated on: Comprehensibility, Imageability, Frequency, Strength of emotional response			×
Knutson et al. [29]	Text	312	All stimuli rated on: Wrongness, Harm, Benefit to actor, Benefit to others, Premeditation, Legality, Social rule violation	All stimuli rated on: Valence, Arousal, Sociality, Frequency, Personal familiarity, General familiarity	×	×	×
Lotto et al. [28]^c^	Text	75	All stimuli rated on: Whether rater would perform sacrifice, Acceptability of performing sacrifice	All stimuli rated on: Valence, Arousal			×
SMID	Images	2,941	All stimuli rated on Wrongness, Care, Fairness, Ingroup, Authority, Purity	All stimuli rated on: Valence, Arousal	×	×	×

^a^Each stimulus in the Chadwick et al. set was rated only on the single dimension it was assumed to be relevant to.

^b^The Clifford et al. set also includes eight factor-analytically-derived dimensions based on wrongness judgments, which map onto the six moral foundations (including Liberty, and with three variants of harm).

^c^The Lotto et al. set consists of sacrificial dilemmas which we tentatively classify as having negative moral valence.

#### Challenge 1: Text-bound morality is inherently limited

In the real world, morality is not limited to a single modality; a wide range of mediums can elicit moral evaluations, from spoken and written words, to images, videos, and actual social interactions. To the extent that the moral psychology literature is built predominantly upon any single medium, it can provide only a partial understanding of morality. Unfortunately, nearly all existing moral stimulus sets are text-based (the lone potential exception being the Geneva Affective Picture Database [2], discussed in further detail in S1 Text).

Unsurprisingly, most studies rely on text stimuli (e.g., see Table 1 of [32] and Table 1 of [33]). The near exclusive reliance on text imposes two constraints on the range of possible study designs. The first constraint concerns the time and effort required for participants to read a large set of vignettes. To present rich stimuli resembling everyday moral phenomena, researchers have few alternatives to detailed vignettes that require sustained concentration to process. The use of such vignettes raises a dilemma for researchers, in which they must choose between either protracted testing durations if the stimulus set is large, or reduced validity and statistical power if the stimulus set is small [34–38] (regarding the latter, statistical power can asymptote well below 1 when stimulus variability is high and the number of stimuli is low [36]). The time constraints imposed by text-based stimuli are especially pertinent in neuroimaging studies in which, for example, presenting a single sacrificial dilemma can require over half a minute of scanning time (e.g., [39,40]), compared to the mere seconds required to present an image stimulus ([15,41]).

A second constraint imposed by text-based stimuli is that such stimuli can be difficult to transport into paradigms that target rapid affective or intuitive psychological processes that are central to many current theories of moral cognition [40,42–47]. Although researchers can potentially employ such paradigms with the presentation of one or a few words [48–51], this comes at the expense of the richness and realism that can be achieved with other mediums (consider the vastly different psychological experiences elicited by seeing the word “assault” as opposed to seeing an image or video of assault, let alone witnessing assault in person).

Moreover, studies using many single-word stimuli may require matching on several potentially confounding factors (e.g., length, frequency). Given the finite number of words in a language however, a suitable set of word stimuli simply may not be identifiable [52]. This is far less of a concern for images. Whereas the English lexicon is estimated to contain around one million words [53], a Flickr search for the term “house” currently returns around 3.5 million unique images. Furthermore, irrelevant features of image stimuli can often be modified to reduce confounds (for example by cropping, altering color composition, etc.), whereas analogous modifications are far less feasible for word stimuli [52].

Beyond these practical considerations, researchers must also consider the possibility that text-based stimuli may be processed differently to non-textual portrayals of the same content (e.g., [54,55]). According to Construal Level Theory (CLT [56,57]), presenting words (vs. images) promotes a high-level construal [58,59]. In the context of moral judgments, recent studies suggest that high-level construal may be associated with greater attention to ends vs. means [60], greater value-behavior consistency [61], and emphasis on different moral values [62–64]. Thus, the CLT literature suggests that presentation medium may systematically influence multiple aspects of moral cognition and behavior. Accordingly, overreliance on any single medium (here, text) may inadvertently distort theoretical accounts of moral psychological phenomena.

Developing a stimulus set that permits rapid presentation of rich, realistic moral content (and differs from the currently dominant medium) would thus help address significant challenges facing much existing moral psychology research. Of the forms such a stimulus set could take, arguably the most versatile medium is images. Image stimuli have a long history in moral psychology [65–68] (apparently even predating sliced bread [69]), offering a set of unique benefits over text-based stimuli. Images can be used in populations where linguistic stimuli are problematic [66,70]. Moreover, images can often be used similarly to text-based stimuli in explicit (or active) paradigms in which participants have their attention directed towards, and are required to deliberate upon, the moral content of the stimuli (e.g., sacrificial dilemmas). Unlike text-based stimuli however, images can also readily be used in implicit (or passive) paradigms [15–17,50,71–73], without sacrificing richness. For these reasons, we chose images as our preferred modality.

#### Challenge 2: Morality is diverse

At present, there exists a large, historically rich literature characterizing the various ways in which people differ in their moral concerns, and in which situations differ in their moral content. Within existing moral psychology stimulus sets however, far less attention has been paid to systematic variation in moral content. Accounting for diversity in the content of moral stimuli is critical, given recent findings that the processing of different kinds of moral content has been shown to (1) recruit, require, or be moderated by, different psychological processes (e.g., [63,74–76]), (2) result in different patterns of inferences in the context of character judgments [77,78], and (3) be differentially affected by psychopathology [79–82]. This implies that if researchers restrict stimuli to a single domain of moral content (e.g., instances of harm), findings cannot be generalized beyond that moral domain, and certainly cannot be considered representative of morality in its entirety. Likewise, if researchers ignore variation in moral content by treating morality as a homogenous entity, findings will either be substantially noisier, or skewed by whatever unaccounted-for moral content happens to dominate the selected stimuli [83].

To address diversity of moral content, we used Moral Foundations Theory (MFT) as an organizing framework for the initial development and description of the image set [84–87]. MFT posits five purportedly innate foundational moral values: (1) *Care*, concerned with prevention or alleviation of suffering, (2) *Fairness*, concerning identification of cheating and exploitation, (3), *Ingroup*, concerned with self-sacrifice for group benefit and preventing betrayal, (4) *Authority*, concerned with respecting and obeying superiors, and (5) *Purity*, concerned with avoiding pathogens through, for example, regulation of sexual and eating behaviors. A tentative sixth foundation, *Liberty*, has been proposed [88,89], but not yet fully incorporated into the MFT research program (thus, for simplicity, we omitted Liberty from the initial validation process). While we remain agnostic about MFT’s claims regarding innateness or modularity [90–94], the theory nonetheless provides a broad, useful, and widely-used description of the way in which people’s moral values differ, as well as of the situational factors that are likely to reveal those differences.

#### Challenge 3: Morality is complex

Another desirable feature of any stimulus set is that it adequately reflects the complexity of its subject matter. One pertinent limitation of many moral stimulus sets in this regard is that they are constructed on an assumption of discreteness, whereby each stimulus is assumed to represent just one moral construct (e.g., grouping stimuli into separate “harm” and “fairness” categories). As a particularly striking example, the Chadwick et al. stimulus set, which sorts 500 stimuli into 10 discrete categories, classifies the act of “Helping build a home for the needy” as charitable, but neither cooperative nor friendly, whereas “Helping someone find a lost dog” is classified as friendly, but neither charitable nor cooperative (see Table 2 in [27]).

Although assuming discreteness in the moral domain may occasionally be desirable, such an assumption may be problematic for two reasons in particular: (1) different kinds of moral content often covary, and (2) different kinds of moral content may interact when people form moral judgments. In both cases, existing research highlights how assuming discreteness may produce misleading findings.

Regarding the first problem, covariation of moral content, stimuli that fit into discrete moral categories (e.g., impure but harmless) may be the exception rather than the rule [24,49,95]. Instead, stimuli that are judged as violations of one moral norm (e.g., Care) are likely to also be judged as violations of other moral norms (e.g., Purity) [24,26,96] (although see [97,98]). To appreciate the implications of this, it is instructive to consider analogous findings in the emotion literature. It is widely accepted–even by basic emotion theorists [99,100]–that people can, and regularly do, experience mixed emotions [101–103]. Likewise, ratings of basic emotion content (e.g., anger, disgust) are often highly correlated in normative ratings for pictorial [104–107], auditory [108] and word-based [109] affective stimulus sets. If researchers fail to account for mixed emotional experiences, they risk mistakenly attributing findings to one emotion (e.g., disgust) which may in fact be driven by another emotion that reliably co-occurs with the target emotion (e.g., anger; for discussion, see [110,111]). Alternatively, if researchers successfully narrow their focus to situations in which *just one* emotion is elicited in isolation, their findings may only be informative about a narrow subset of emotional experience (for reasons discussed next). The point here is not that different emotions or different kinds of moral content are hopelessly confounded, but rather that content overlap is a challenge that must be tackled head-on to gain a deeper understanding of moral phenomena (e.g., [92,95,97,111]).

Regarding the second problem, many recent studies underscore the importance of considering interactions between different kinds of moral content. For example, evaluations of harmful actions can differ markedly, depending on the perceived justness of the action or the relational context in which it takes place: many people judge self-defence differently than they do unprovoked assault, just as they judge a teacher hitting a student differently than the converse, even if each example entails similar amounts of suffering [112–114]. Additionally, people’s *relative* concern for specific values (e.g., fairness as opposed to loyalty) influence moral judgments (e.g., of whistleblowing [115]). In person perception, people’s valuation of a particular virtue (e.g., dedication) depends on the target’s standing on other virtues (e.g., kindness [78]). Once again, one can look to the emotion literature where responses to emotion-eliciting stimuli are contingent on *combinations* of emotional responses (for example when experiencing sympathy either in isolation or in conjunction with other emotions [116]).

Assuming discreteness of moral content (e.g., that a purported harm violation contains only harm-related content) potentially misrepresents the structure of the moral domain or, at the very least, restricts research to a potentially narrow subset of it. In either case, the discreteness assumption discourages exploration of interactions between kinds of moral content (e.g., [78]), and likely overlooks real-world cues that are frequently relied upon for moral judgment, resulting in misleading conclusions and hampering efforts to decompose the moral domain into its constituent parts (for more general discussion, see [117,118]).

Constructing stimulus sets that overcome this challenge necessitates collecting a broad range of normative data without artificially constraining stimuli to be purportedly discrete instances of different moral content domains (e.g., by having stimuli only rated on the content domain to which they are assumed to belong [27], or by systematically excluding stimuli that map onto multiple content domains [26]). Equally importantly, the complex, multidimensional nature of moral content necessitates the construction of a *large* stimulus set that can accommodate both *systematic* designs that can be used to avoid confounds (to the extent possible [52,119]), and more *representative* designs that broadly sample the moral domain in a way that resembles everyday moral experiences in terms of the prominence and co-occurrence of different moral factors [117].

#### Challenge 4: There is more to morality than immorality

A final challenge that limits most existing moral stimulus sets is that they often reflect the broader trend in moral psychology to segregate moral goods and moral bads into separate research programs. While some theories posit processes or content domains that describe one or the other end of the moral spectrum [113,120,121], others posit processes or content domains that operate across the entire spectrum (e.g., [85,122]). However, the data supporting any one of these theories are typically limited to one pole (often the negative pole). Moreover, when both ends of the spectrum are covered within the same theoretical framework, this is often explored in separate studies with different methods, posing challenges for the integration of findings (one notable exception being [123]). Perhaps the primary reason for this is the scarcity of stimulus sets covering both ends of the moral spectrum.

This is unfortunate for two reasons. First, although various theories of moral psychology employ concepts with substantial overlap (e.g., Humanity [120], Harm/Care [85], and Not harming [122]), the dearth of stimulus sets covering both ends of the moral spectrum presents major obstacles to integrating across theories whose primary concerns and/or evidence bases lie at opposite ends of the spectrum. Second, for theories seeking to describe both ends of the moral spectrum (e.g., [85,122]), the lack of studies *simultaneously* covering both ends hinders efforts to assess whether these theories achieve this descriptive goal. This is particularly important given widespread evidence pointing to differential processing of positive and negative content both in general [124–126], and in the moral domain in particular [126–128]. This suggests that concepts and processes used to describe one end of the moral spectrum may not translate so well to the other. The obvious solution to this challenge is to develop a stimulus set covering the entire moral spectrum.

### Summary and overview of the present studies

The primary aim of the current studies is to develop a large, versatile stimulus set that addresses the critical challenges described above. Specifically, we (1) offer a departure from standard text-based moral stimuli by assembling a large image database containing rich, concrete, realistic stimuli suitable for a wide range of paradigms, (2) provide a diverse stimulus set sampling as much of the moral domain as possible, (3) avoid assuming (or requiring) that each stimulus can be placed in a discrete category (e.g., “fairness violation”) by collecting normative data across the five moral foundations, and (4) provide stimuli that span the entire moral spectrum, from negative to positive, to facilitate comparison and theoretical integration across theories that have thus far been restricted to one end of the moral spectrum.

To do so, we chose an approach rarely used in stimulus set construction: we build our stimulus set from the bottom up, through crowdsourcing, to mitigate potential researcher-selection biases (although the crowdsourced images are supplemented with researcher-selected content). Additionally, we restricted the database to include only images that are Creative Commons licensed (or have similarly permissive licenses), which has the benefits of (1) allowing researchers the freedom to present the materials in both online and offline settings without concern about copyright restrictions (an issue receiving increasing attention with the development of new research materials across various fields [4,14,129,130]), and (2) enabling a range of novel research applications that leverage the wealth of text (and other) data linked to many of the images, as discussed towards the end of the paper.

The remainder of the paper is structured as follows. In Study 1, we report the generation of the image set, covering the sourcing and screening of images. In Study 2, we report the norming of the image set, and provide a detailed description of important features of the image set that we expect to be of interest to moral psychology researchers.

## Study 1

To avoid biasing the content of different moral dimensions, we opted for a bottom-up approach for image collection, crowdsourcing most images rather than collecting them ourselves. To this end, Study 1 reports the image collection and screening procedure.

### Method and results

All participants for Study 1 were recruited via Amazon’s Mechanical Turk (AMT), an online crowdsourcing platform where people perform tasks referred to as Human Intelligence Tasks (HITs) in exchange for monetary compensation [131–133]. We restricted eligibility to participants with approval ratings ≥ 95% and ≥ 1,000 previous tasks completed.

#### Ethics statement

This study received ethical clearance from the University of Melbourne (ethics ID 1341310). Prospective participants were directed from AMT to an online information sheet describing the study, after which participants provided informed consent if they wished to participate.

#### Image collection

To generate a pool of candidate images, we recruited 476 participants into an image collection task. Exactly half of the participants were female, and a substantial majority (around 90%) reported having completed or commenced at least some university or college education. Participant ages ranged from 18 to 67 (*M* = 33.26, *SD* = 9.85). No other demographic information was recorded. To increase participant diversity, around 10% of participants were recruited from India, with the rest from the United States.

Each participant was asked to search for and provide URLs for 20 images, that were available from the Wikimedia Commons or Flickr, and that they believed were representative of two randomly assigned moral concepts (i.e., 10 images per concept). Concepts were both positively and negatively valenced, and spanned a wide range of moral content. While some of the concepts were directly related to those used in moral foundations research, we also included a number of non-MFT concepts (e.g., “Deception,” “Self-Control” and generic morality / immorality concepts). To increase the diversity of the images, we adopted different strategies across iterations of the task, such as altering the concreteness of the moral concepts (e.g., “People behaving immorally” vs. “Unfairness” vs. “Theft”), and having participants generate their own search prompts related to each moral concept (a full list of moral concepts is provided in Table 2, and full search instructions provided in S2 Text).

**Table 2:** Image search concepts used in image set generation.

Animals behaving immorally	Hedonism	Reminders that humans are animals too
Animals behaving morally	Hierarchical Relationships	Respect / Subversion
Benefitting one’s own group at the expense of others	Hierarchy in the animal kingdom	Rudeness
Care / Harm	Inequality	Sacrifice for the good of one's group
Compassion in the animal kingdom	Injustice	Sanctity / Degradation
Conformity	Lack of Self Control	Self-Control
Contamination	Liberty / Oppression	Selfishness
Cooperation / group behavior in the animal kingdom	Loyalty / Betrayal	Social Hierarchy
Cruelty	Maintaining boundaries between groups	Teamwork
Cruelty in the animal kingdom	Naturalness	Theft
Deception	Obedience	Tradition
Discrimination	People behaving immorally	Treachery
Equality	People behaving morally	Un-naturalness
Exploitation	Politeness	Unfairness
Fairness in the animal kingdom	Proportionality (i.e. getting what one deserves)	Untrustworthiness
Fairness/Cheating	Proportionality (i.e. getting what one has earned)	Wholesomeness
Generosity	Reciprocity (i.e. paying back in kind)	

After excluding duplicate URLs, corrupted or irretrievable images, and images that were smaller than 640 by 480 pixels, this process yielded 4,092 images. An additional 362 researcher-contributed images were added to the pool after reaching a saturation point where later participants frequently returned images that had already been submitted by previous participants. This increased the total to 4,454 images.

#### Image screening

Next, we programmatically collected metadata (including licensing information, image author, title etc.) for all retrievable images using the Wikimedia and Flickr application programming interfaces (APIs). To ensure the final image set could be used as widely as possible, we retained only Creative Commons (or similarly permissively) licensed images. Filtering out images with more restrictive licensing left a pool of 3,726 images.

**Fig 1:**
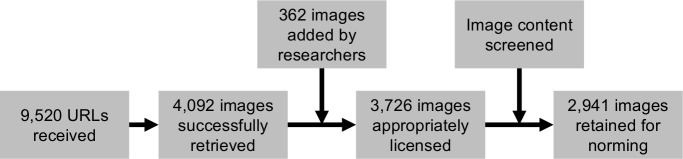
Summary of database construction process.

An independent AMT sample of 285 AMT workers (48% male; age *M* = 35.72, *SD* = 10.75) screened the remaining images for various features so that we could select a subset of images most useful for a wide range of research applications. Images were excluded if they (1) contained famous people, (2) prominent text (such that extracting the meaning of the image required reading), (3) watermarks or commercial logos, or (4) were non-photographic (e.g., cartoons). Each participant screened around 60–70 images, and each image was screened by at least five participants. Features were coded as present if a majority rated them as such. Through this process, we retained 2,941 eligible images to be subjected to further rating. A summary of this process is presented in Fig 1. Additionally, participants coded images for the presence of people (appearing in 63% of images in the final pool), animals (17%), and landscapes (15%) so that such features can be incorporated into stimulus selection procedures (coding of additional features is underway).

## Study 2

Having generated a pool of images in Study 1, Study 2 involved collecting normative ratings for the images on a set of moral and affective dimensions.

### Method

#### Ethics statement

This study received ethical clearance from the University of Melbourne (ethics ID 1341310). As in Study 1, prospective participants were directed to an online information sheet describing the study procedure, after which they provided informed consent if they wished to participate.

#### Participants

We recruited a large sample from two sources: AMT (as in Study 1), and the University of Melbourne undergraduate psychology research participation pool. For AMT participants, eligibility was restricted to workers located in the United States with approval rates ≥ 90%, and ≥ 100 previously approved HITs. After excluding participants using an extensive set of criteria to detect inattentiveness (detailed in S3 Text), our final sample comprised 1,812 AMT participants (49% male; *M*_age_ = 36.75, *SD*_age_ = 11.20), and 904 undergraduate participants (24% male; *M*_age_ = 19.31, *SD*_age_ = 3.48) who, combined, provided a total of 820,565 ratings.

Sample size was determined based on a target of obtaining at least twenty ratings for each image on each dimension, although the average number of ratings was considerably higher (*M* = 34.88). Such a number of ratings per image is comparable to existing affective image sets, especially considering the comparatively larger number of images and dimensions that were rated (see S1 Text for comparisons of the SMID with existing affective image sets regarding rating frequencies). To ensure that ratings were not skewed by a lack of moral or political diversity, we ensured that each image was rated on each dimension by a minimum of five AMT participants each self-identifying as liberal, conservative, or moderate/other (and at least five Australian undergraduate participants).

#### Materials and procedure

All 2,941 eligible images were rescaled to a height of 400 pixels (maintaining their original aspect ratios), and then randomly split into 99 largely non-overlapping batches of 30–40 images. Two images (one of a thunderstorm, and one of the scales of justice) were deliberately included in all batches to provide a common context and reduce the likelihood of some batches (by chance) including highly idiosyncratic moral content. Additionally, we discovered a small proportion of images that appeared in multiple batches because, during Study 1, participants occasionally submitted different URLs indexing the same image. Because these images were identified *after* commencing the study, ratings for these duplicate images were combined *post hoc*.

In later stages of data collection (i.e., for most of the Australian undergraduate sample), we implemented a strategy to obtain additional ratings for images eliciting highly variable responses. Specifically, we constructed a pool of images whose normative ratings had the largest standard errors. For participants rating batches containing < 40 images, additional images were drawn from this pool until that participant had been assigned a total of 40 images.

After providing informed consent, participants were randomly assigned to rate one batch of images via their web browser in a custom-coded JavaScript task (*N* = 23–34 participants per batch). Images were rated on each of the following eight dimensions (each on a 1–5 scale, using the keyboard): valence (“unpleasant or negative” to “pleasant or positive”), arousal (“calming” to “exciting”), morality (“immoral/blameworthy” to “moral/praiseworthy”), and the five moral foundations, Care, Fairness, Ingroup, Authority and Purity. When rating images with respect to moral foundations, participants rated the extent to which the images made them think about that specific foundation (“not at all” to “very much”). Rating dimension labels are summarized in Table 3. Before rating images on a dimension, participants read a detailed description of that dimension (provided in full in S4 Text). Participants rated all images in the assigned batch on one dimension before proceeding to the next dimension, until all dimensions had been rated. Image and dimension order were randomized within participants to prevent order effects.

**Table 3:** Image rating dimension summary.

Dimension	Label	Lower Anchor	Upper Anchor
Valence	This image is …	UNPLEASANT or NEGATIVE	PLEASANT or POSITIVE
Arousal	This image is …	CALMING	EXCITING
Morality	This image portrays something …	IMMORAL / BLAMEWORTHY	MORAL / PRAISEWORTHY
Harm	This image makes me think about the concept of CARE / HARM	NOT AT ALL	VERY MUCH
Fairness	This image makes me think about the concept of FAIRNESS / CHEATING	NOT AT ALL	VERY MUCH
Ingroup	This image makes me think about the concept of LOYALTY / BETRAYAL	NOT AT ALL	VERY MUCH
Authority	This image makes me think about the concept of RESPECT / SUBVERSION	NOT AT ALL	VERY MUCH
Purity	This image makes me think about the concept of SANCTITY / DEGRADATION	NOT AT ALL	VERY MUCH

After completing the rating task, participants were redirected to a questionnaire in which they provided basic demographic information (including political orientation), and completed the 30-item Moral Foundations Questionnaire (MFQ [134]). Analyses of these self-report data are to be reported elsewhere.

## Results and discussion

Here, we present a multifaceted assessment of data quality, followed by a high-level summary of image-level variability within and across dimensions. (Note that we defer discussion of general recommendations for use until the General Discussion.)

### Inter-rater consensus

First, we sought to quantify the degree of consensus in the ratings for each dimension. One important motivation for such analyses is that it is unclear what degree of consistency to expect when eliciting single-item ratings of broad, abstract moral content dimensions (given the lack of previous research addressing the question). To this end, we computed two variants of the intra-class correlation coefficient (ICC) separately for each dimension in each batch of images. ICCs are commonly interpreted as reflecting the proportion of variance in ratings attributable to the target (here, image stimuli) [135–137]. As such, higher values indicate greater consensus arising from such factors as common (1) interpretations of the rating scale, (2) interpretations of the images, or (3) scale use.

**Fig 2:**
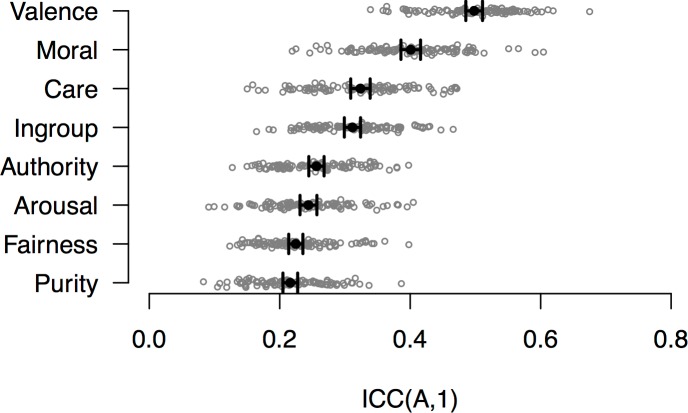
Distributions of intra-class correlation coefficients for each rating dimension across image batches. Grey points represent individual observations (i.e., each of the 99 batches). Black points represent the average ICC(A,1) across all batches, with error bars representing 95% CIs.

Using the *irr* package for *R* [138], we first computed ICC(A,1) for each batch, where (1) target (image) and rater (participant) were both treated as random effects (see [136]), (2) coefficients were calculated based on absolute agreement (rather than consistency), and (3) the coefficient reflects the reliability of a *single* rating. The distribution of ICCs across batches for each dimension, across the entire sample, is presented in Fig 2. Note that ICCs calculated based on absolute agreement (i.e., ICC(A,1)) will tend to be lower than ICCs calculated based on consistency (i.e., ICC(C,1)), as indeed was the case here: across all dimensions and batches, the average ICC(A,1) was about .04 less than the average ICC(C,1).

As shown in Fig 2, there was substantial variability in consensus across rating dimensions, with valence (and to a lesser extent, morality) eliciting the greatest amount of agreement, and the five moral foundations eliciting relatively less. Importantly, moral foundation content had ICCs comparable to that of arousal. Amongst the five moral foundations, Care was the most agreed upon dimension, and Fairness the least.

Compared to frequently cited rules-of-thumb [139], these reliabilities range from “fair” (.40 ≤ ICC < .60, for valence and morality) to “poor” (ICC < .40, for all other dimensions). However, it should be noted that these guidelines were intended for the evaluation of clinical assessment instruments (which often comprise multiple items). Moreover, to our knowledge, ICCs of any kind are neither reported for validation studies of existing affective image sets nor textual moral stimulus sets, making it difficult to provide a sufficiently similar reference point for comparison. Finally, we note that ICC(A,1) is insensitive to the number of ratings obtained per image, and thus does not reflect the reliability of the norms, but rather of a single rating (however, the second variant of the ICC reported below does take rating frequency into account). Nonetheless, the fact that a large proportion of the variance in image ratings is explained by sources *other than* the image itself suggests, perhaps unsurprisingly, that factors such as people’s idiosyncratic interpretations of moral concepts (and the stimuli themselves) exert substantial influence on ratings (we return to this point below).

#### Precision of measurement

**Fig 3:**
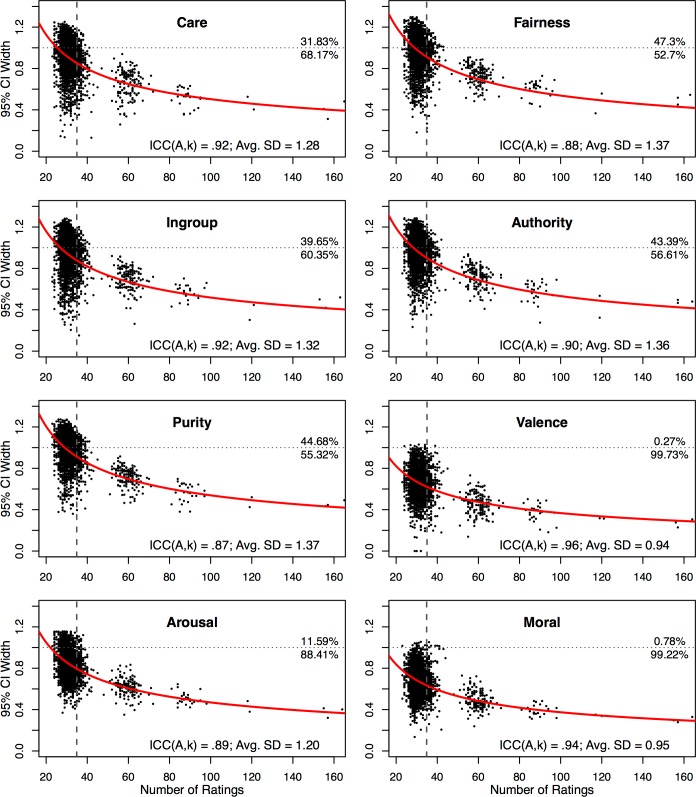
Scatterplots of image norm 95% confidence interval (CI) widths as a function of the number of ratings, by dimension. Each point represents an individual image. Vertical axis represents 95% CI width (in scale points) for each image, with images lower on the axis having more precise measurement. Horizontal axis represents the number of times each image has been rated. Red curve represents expected 95% CI width given the average rating SD (inset) for that dimension and number of ratings. Vertical dashed grey line represents average number of ratings per image for that dimension. Horizontal dotted grey line marks a 95% CI width of 1, with the percentage of images falling above or below this threshold presented at the right end of the line. ICC(A,k) (inset) represents the average ICC(A,k) across batches.

Next, we examined the degree of *precision* in the image norms which, unlike the analyses above, is not just a function of inter-rater consensus, but also of the amount of data collected (given that noisy but unbiased measures will give accurate estimates with enough observations). To measure precision, for each dimension, we used two metrics. First, we computed the expected width of the 95% confidence interval for an “average” image as a function of (1) rating frequency, and (2) the average of the standard deviations (SD) of ratings for all 2,941 images on that dimension. Expected CI widths for each dimension at various rating frequencies (measured in scale points for a five-point scale), are shown in Fig 3, along with observed CI widths for each image. Fig 3 thus shows (1) the accuracy of norms across the image set, as well as (2) the expected gain in precision if more data were to be collected. Additionally, we computed ICC(A,k) (also displayed in Fig 3), providing a measure of reliability of image norms created by averaging across raters.

As shown in Fig 3, we achieved a 95% CI width of less than one scale point (i.e., plus or minus half a scale point) for most images on most dimensions. More concretely, this means that if one wished to sample images that were typically perceived in a specific way (e.g., as highly immoral), the amount of data available allows researchers to do so with a reasonably high degree of confidence. Additionally, we note that averaged ratings on all eight dimensions achieved “excellent” reliability (ICC ≥ .75, based on the guidelines proposed in [139]). Although additional data would further enhance precision, such would only be achieved with diminishing returns for every additional participant: halving the average CI width (particularly for the moral content dimensions) would effectively require increasing the rating frequency by a factor of around four or five (requiring around 10,000 to 13,000 raters, given the current task parameters).

#### Moral content distributions

**Fig 4:**
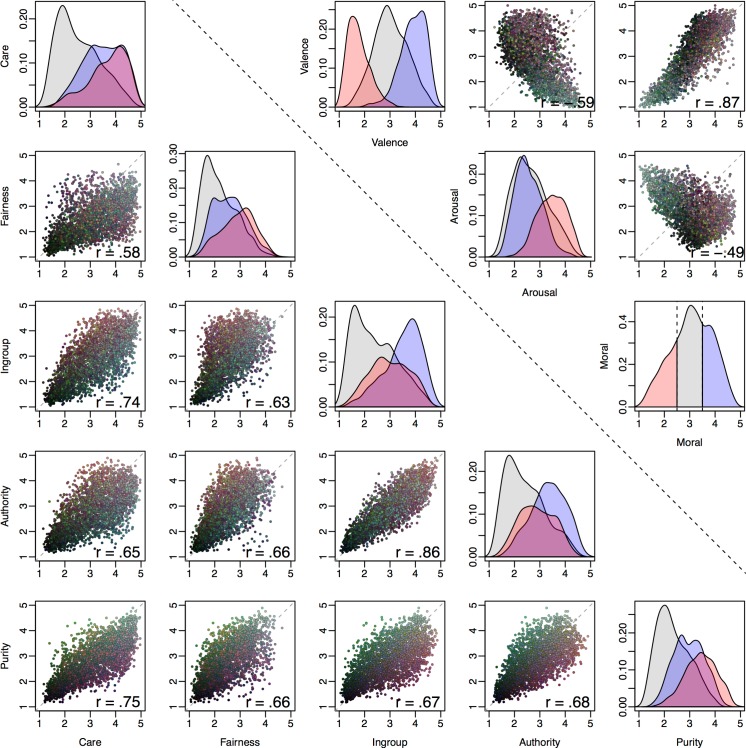
Correlations and rating distributions across images for (1) moral content dimensions (lower section below black dashed line), and (2) valence, arousal and morality (upper section above black dashed line). On diagonal: density plots of relevance ratings for each moral foundation (lower section), and valence, arousal and morality (upper section), with each plot divided into morally good (blue; mean moral rating > 3.5), bad (red; mean moral rating < 2.5), and neutral (grey; all other images). Off diagonal: scatterplots of average ratings for all images with Pearson correlation coefficients inset. To aid interpretation, point color represents moral content ratings collapsed into the CAD triad [140] with each of the three dimensions mapping onto a different color (Community [Ingroup + Authority] = red; Autonomy [Care + Fairness] = blue; Divinity [Purity] = green).

Having explored the reliability and precision of the ratings, we next describe the distribution of moral content ratings, beginning with univariate and bivariate distributions of each moral content dimension (and pairs thereof), depicted below in Fig 4. Depending on researchers’ goals or assumptions, an ideal image set might contain images spanning all possible values for each dimension (and combinations of dimensions), such that researchers could easily select images meeting arbitrary criteria (e.g., high on dimension A, low on dimension B, etc.). However, as has been repeatedly demonstrated for affective stimulus sets, this combinatorial goal is difficult to achieve in practice (e.g., finding negatively valenced, low-arousal stimuli [1,2,4,12]). As shown in Fig 4, a similar pattern obtains for moral content (and for valence and arousal).

Based on a strict “modular” view of moral foundations as discrete domains, one might *intuitively* expect relevance ratings for the five dimensions to be at most weakly or moderately correlated (even if a modular view does not strictly require this). Fig 4, however, shows that all five foundations were strongly positively correlated (all *r*s > .5, all *p*s < .001), suggesting that relatively “pure” instances of individual foundations (i.e., scoring highly on one, but low on all others) may be somewhat rare, as suggested in the Introduction and by previous research [26,141] (and mirroring findings in the basic emotion literature).

More broadly, we caution that these correlations ought not to be taken as refuting the existence of discrete moral “modules” for two reasons (although similar correlations have been interpreted as such elsewhere [24,142]). First, the fact that two variables are strongly correlated does not necessarily imply that they are the same thing (e.g., consider height and weight in humans which, in two large datasets available from http://wiki.stat.ucla.edu/socr/index.php/SOCR_Data, exhibit correlations > .5). Second, the correlations reported in Fig 4 were observed at the group level (aggregated by image). It is entirely possible for analogous correlations within individuals to differ substantially [143–145]. For example, although the image-level correlation between Care and Fairness relevance was .58, when one computes the Care-Fairness correlation within each individual, the average correlation is .32, and the correlation is in fact negative for 11% of participants.

#### Can foundation-specific images be identified?

**Fig 5:**
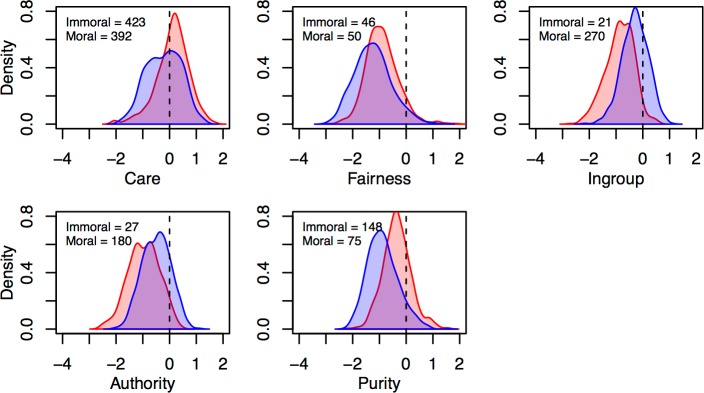
Density plots of image uniqueness score distributions for each moral foundation by moral valence. Morally good images (blue) are defined as having mean moral ratings > 3.5, and morally bad images (red) as having mean moral ratings < 2.5. Number of morally good and bad images per foundation with uniqueness scores > 0 inset.

Although images that *exclusively* represented specific foundations were rare, it is possible to identify images that relate more strongly to one foundation than others. To identify such images, we devised a set of *uniqueness scores* for each image on each foundation (included in the normative data available at https://osf.io/2rqad/). Uniqueness scores were computed by taking an image’s score on a given foundation, and subtracting from this value the maximum score the image received on the other four foundations (alternative methods, included in the norms, but omitted here for brevity, are described in [6,109]). For example, consider an image for which the average relevance to Care = 5. If that image’s highest average score on the other foundations is Purity = 3, we assign a Care uniqueness score of 5 − 3 = 2. A positive uniqueness score of *x* for a given foundation thus indicates that an image is on average rated at least *x* scale points higher on that foundation than all foundations. Uniqueness score distributions for each foundation are summarized in Fig 5.

As shown in Fig 5, uniqueness scores tended to cluster around or below zero (unsurprisingly, given that by definition, uniqueness scores for each image will be ≤ 0 on four of five dimensions). While the maximum possible uniqueness score was 4 (5–1), few images scored above 2 for any dimension. Importantly, however, the image set included at least 46 morally good and 21 morally bad images with positive uniqueness scores for each individual foundation (i.e., ≥ 46 morally good Care images, ≥ 46 morally good Fairness images, and so-on), indicating the presence of images predominantly (if not exclusively) depicting each foundation. Moreover, when one visually inspects the images with high uniqueness scores there is a high degree of face validity for the images representing each moral foundation.

#### Mapping moral content onto valence, arousal and moral judgments

**Fig 6:**
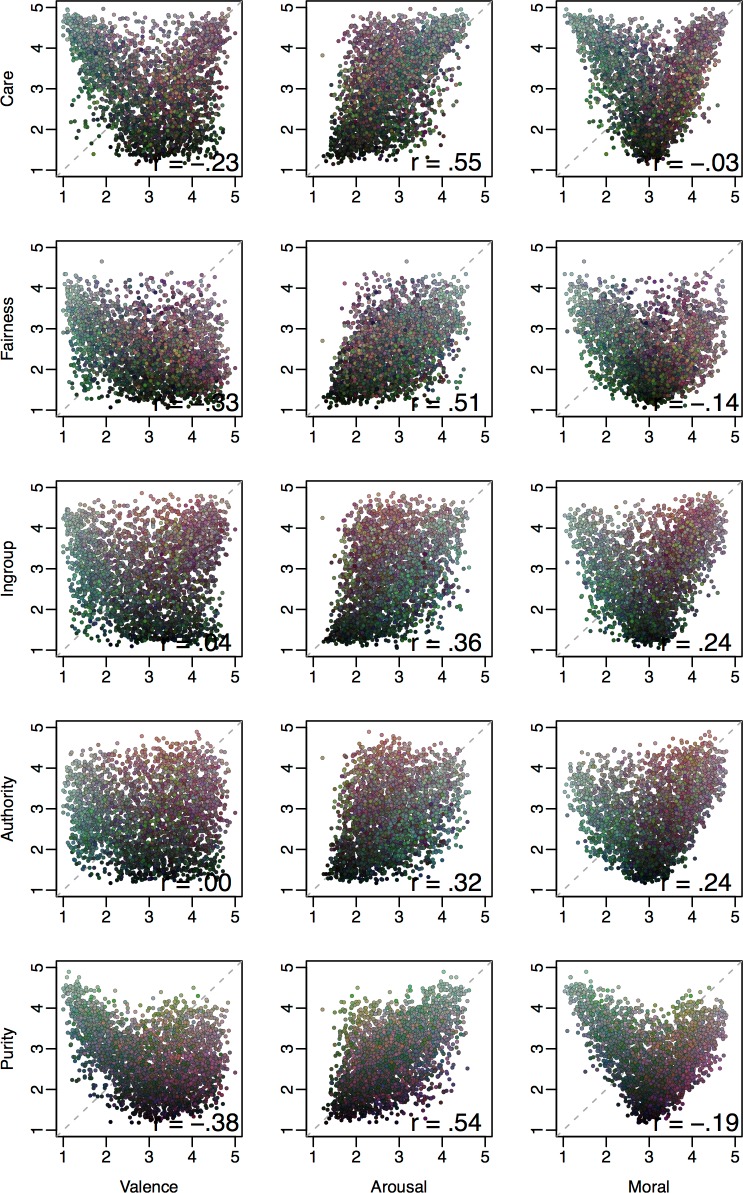
Correlations between moral content dimensions and valence, arousal and morality ratings across images. Each cell contains a scatterplot of average ratings for all images for all moral foundations with valence, arousal and morality; To aid interpretation, point color represents moral content ratings collapsed into the CAD triad [140] with each of the three dimensions mapping onto a different color (Community [Ingroup + Authority] = red; Autonomy [Care + Fairness] = blue; Divinity [Purity] = green).

Two broad questions that have motivated much research in moral psychology concern (1) the relative importance of different moral content domains for explaining moral judgments [49,121,141,146] and (2) links between moral cognition and the core affective dimensions of valence and arousal [45,147–152]. Here, we describe image-level correlations between moral content ratings on the one hand (i.e., relevance to each of the five moral foundations), and moral judgments, valence and arousal on the other. It should be noted however that, as image-level correlations, one should not assume that analogous correlations hold within (or between) individuals. Rather, these correlations reflect the content of images as they tend to be perceived by groups (which may nonetheless serve as plausible *hypotheses* regarding within- or between-person correlations). These correlations are presented in Fig 6.

As can be seen for judgments of morality (Fig 6, right column), the bivariate distributions resembled a clear v-shaped relationship, such that images receiving extreme moral judgments (either positive or negative) were rarely rated as irrelevant to any of the moral foundations. This pattern was most pronounced for Care and Purity.

Recall that the moral content variables were coded on a non-valenced scale, ranging from irrelevant to highly relevant to a specific content domain (e.g., with Harm/Care *both* anchoring the upper-most response), rather than a valenced scale (with Harm and Care on opposite poles). Thus, the v-shaped pattern emerged as a predictable consequence of images with extreme moral content on a specific dimension tending to elicit positive or negative moral judgments depending on whether the image portrayed the positive or negative pole of that content domain.

The pattern of findings was similar for valence (Fig 6, left column), though with a less prominent (and less symmetric) but still noticeable v-shaped pattern for Care and Purity. The somewhat asymmetric pattern suggests that whereas negatively valenced images tended to be loaded with Care and/or Purity content, this was less often the case for positively valenced images (at least for the images included in the database).

No such v-shaped relationship was apparent for arousal. Instead, all five content dimensions were positively correlated with arousal (especially Care, Fairness and Purity), suggesting that low-arousal images were relatively devoid of moral content, whereas highly arousing images were more likely to be rated as containing various kinds of moral content.

#### Exploring within-dimension variability

**Fig 7:**
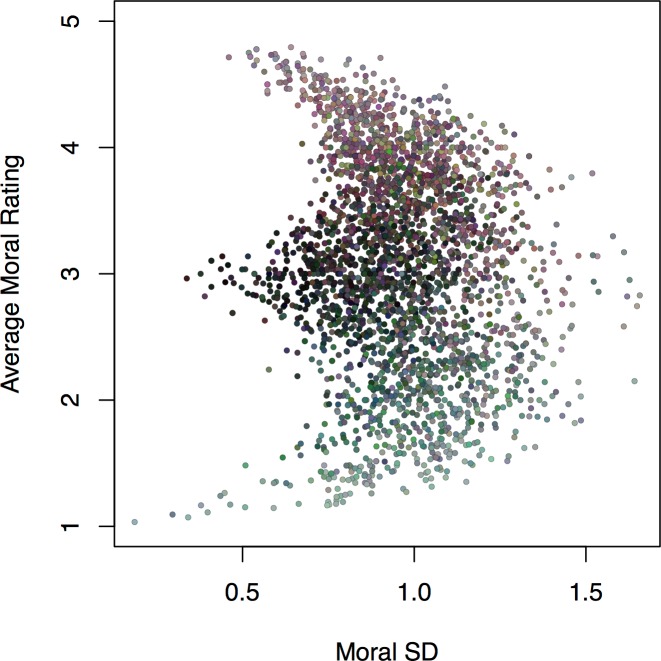
Scatterplot of moral rating standard deviation against moral rating mean for each image. To aid interpretation, point color represents moral content ratings collapsed into the CAD triad [140] with each of the three dimensions mapping onto a different color (Community [Ingroup + Authority] = red; Autonomy [Care + Fairness] = blue; Divinity [Purity] = green).

Regardless of one’s research goals, an important (but neglected) consideration in stimulus selection concerns stimulus-level variance (e.g., whether an image tends to elicit uniform responses or strong disagreement with regards to some feature). Users of the SMID may benefit from looking beyond simple averaged ratings by purposefully selecting images with levels of variability that match one’s aims. As shown in Fig 7, using moral ratings as an example, images elicited a wide range of variability in their ratings, with some images eliciting nearly uniform judgments (with SDs at or around 0), and others eliciting substantial variability (SDs of around 1.5 scale points). Moreover, this was the case for images across the moral spectrum.

Rather than simply reflecting random noise in the ratings, we suggest that rating variability can be meaningfully accounted for by at least three separate substantive sources (all of which would be expected to produce higher SDs, and could in principle be clearly separated with additional measurements).

The first and most straightforward source is ambiguity, whereby images invite multiple interpretations (e.g., an image that could plausibly be construed as either play-fighting or assault), or may simply be difficult for viewers to interpret (e.g., because of high visual complexity). Rather than being an altogether undesirable quality, ambiguous stimuli may be ideally suited for many kinds of paradigms (e.g., [48,153–157]).

The second source of variability, reflecting intrapersonal processes, is ambivalence. People can simultaneously hold positive and negative evaluations of both moral [158] and non-moral [159–161] stimuli, which would result in greater variability in judgments (see [159]). Again, rather than reflecting an altogether undesirable feature of the image set, ambivalence-inducing images may prove useful for (among other applications) probing the integration of conflicting moral information [158]. Although the data presented here cannot speak to the presence of ambivalence, such can easily be measured by adapting measures such as the evaluative space grid [162].

Finally, we consider a third source of variability operating at the interpersonal level: divisiveness. Given differing moral concerns, people may simply disagree with each other regarding their moral evaluation of an image, absent any disagreement about what is portrayed in it (i.e., absent any ambiguity). Once again, divisiveness may prove highly useful for specific research goals such as eliciting psychological or physiological responses that are diagnostic of one’s political or moral preferences (e.g., [41,72,163]), or developing pictorial measures of individual differences [41,65,66,130,156,164–166]. In S5 Text, we present preliminary analyses that attempt to empirically identify images that are divisive with respect to political orientation and gender.

## General discussion

Methods and materials are a driving force behind scientific progress [167]. The primary aim of this project was to expand the range of tools available to moral psychology researchers by developing and validating a novel image database. In this final section, we briefly discuss (1) important gaps in the literature that the SMID can address, (2) potential applications of the image set, accompanied by some general guidelines for use, (3) potential extensions and finally, (4) limitations of the database.

### Improvements over existing stimulus sets

One of the more obvious advantageous features of the SMID is its size and scope: it is the largest freely available moral stimulus database assembled to date, and one of the few that covers both the morally good and bad poles of a range of content dimensions. Moreover, unlike existing stimulus sets, the database is not limited to the portrayal of moral actions, but also contains images of objects and symbols that can also be the target of moral evaluations, and are worthy topics of study in themselves [168–170].

The size of the SMID gives rise to two particularly important benefits. First, in populations in which non-naïveté may be a concern, such as AMT [171,172], the size of the stimulus set reduces the likelihood of participants repeatedly encountering the same stimuli over multiple studies by different labs. Furthermore, in light of salient concerns within the field of psychology regarding reproducibility [173–175], the size of the image set enables researchers to address the underemphasized issues of (1) stimulus (re)sampling in replication efforts [176–178], and (2) sampling a sufficiently large number of stimuli to achieve sufficient statistical power to account for stimulus sampling variance (relevant in psychology as a whole [36], but especially pertinent in resource-intensive neuroimaging research [38,179]).

Beyond its size and scope, there are many ways in which the SMID is qualitatively different to currently available stimulus sets. Among the most prominent of these differences is the SMID’s reliance on images (rather than text) which allows researchers to run studies that would be impractical with text-based stimuli (e.g., [163]). Perhaps the greatest benefit of the SMID over existing stimulus sets, however, is that of greater ecological validity. While one of the most commonly used tool in moral psychology (sacrificial dilemmas) has been criticised for a severe lack of ecological validity [180], the SMID contains detailed depictions of real-world actions, objects, scenes and situations.

The SMID is also among the only databases in which mixed moral content is explicitly modelled. We have departed from the common practice of constraining each stimulus to map onto one and only one content domain [26,27], instead favouring an approach that embraces the complexity of moral phenomena. Such an approach minimizes the impact of theoretical assumptions on the composition of the stimulus set, making it ideally suited to analytic approaches designed to accommodate complex, multidimensional stimuli [181–183], and allowing researchers to explore relatively neglected research topics concerning, for example, interactions between moral content domains [78,112].

Finally, although the SMID is first and foremost an image set, it is also unique in that it spans multiple mediums, with linked text data available for a substantial proportion of the images in the form of Wikipedia pages and Flickr tags or comments (and in many cases, other web pages that also use the same images). Thus, one intriguing application of the image set entails leveraging the enormous quantities of text and metadata in webpages containing these images using the many available methods (e.g., [184–190]). Much as previous research has attempted such feats as using linguistic data to estimate people’s values [191,192], documents’ value content [21,193–197], or the affective connotations of words [198,199], so too could researchers attempt to estimate the moral content of images based on linked text data (for an impressive demonstration of this approach, in combination with computer vision techniques and applied to emotion recognition, see [200]).

### Applications and recommendations for use

In addition to the recommendations provided throughout the paper, here we offer some additional guidelines for researchers intending to use the SMID in experimental research.

#### Selecting optimal subsets

Selecting an optimal set of stimuli can be a highly complex challenge [52,201,202]. In particular, sampling an optimal set from a larger pool becomes increasingly labor-intensive as either the size of the pool, the size of the sample, or the number of variables to be controlled, increases [52]. Thus, if one considers (1) the size of the SMID, (2) the number of stimuli required to run a well-powered study (see [36]), (3) the large number of variables for which normative ratings are available in the database, and (4) debates within moral psychology regarding the effects of various confounds on existing findings (e.g., [24,203–207]), manually selecting subsets of stimuli will produce far-from-optimal solutions for all but the simplest stimulus selection problems. Thus, systematic approaches to stimulus selection are of great importance. Fortunately systematic methods and software packages for stimulus selection are readily available [201,208–210]. To facilitate the adoption of systematic approaches to stimulus selection within the SMID, we have written a generic stimulus selection script for use with the SOS toolbox for MATLAB [201], along with a generic image rating task script programmed using the Python library PsychoPy [211]. Both are scripts available at https://osf.io/2rqad/, and can be easily modified to accommodate researchers’ own research needs (for users without access to MATLAB, a standalone executable version of SOS is also available).

Similarly, in many paradigms, low-level visual features (e.g., luminance or contrast) or high-level visual features (e.g., the presence or absence of human faces) may produce unwanted confounds that undermine the validity of findings (e.g., [12,212–214]). Indeed, as shown in S1 Table, we observe weak though nonetheless significant associations between some visual features and content dimensions. Consideration of such factors is especially pertinent in neuroscientific investigations of moral cognition where researchers generally wish to avoid inducing differences in brain activity with confounded, non-moral features. Fortunately, such features can often be quantified and incorporated into the stimulus selection procedures described above. When control via selection proves difficult, low-level features can be manipulated using readily available software [215]. Additionally, manipulations of high-level image features (e.g., object transfiguration) are becoming increasingly feasible [216,217], presenting fascinating directions for future experimental research. However, for researchers manipulating any aspect of images to achieve statistical control, we urge caution given that modifying seemingly irrelevant perceptual features may influence affective [218,219] and moral processes [219–225].

#### Maximising reproducibility

The primary motivation for developing the SMID was to facilitate rigorous, efficient, and cumulative moral psychology research. Here, we briefly discuss how the database can best serve these goals. To meet the minimum standards for reproducibility, we first recommend that researchers list the unique identifiers of all images selected for their studies. Second, where stimuli are programmatically sampled, sharing code used to sample images will enable replications with different stimuli selected under exactly the same sampling regime [176]. Finally, an emphasis on data sharing offers perhaps the most productive step researchers can take when using the SMID. Given that each image has been normed on multiple dimensions (and that this set will continue to expand), data generated using the SMID has great potential for reuse beyond the original question(s) motivating a given study. Given the potential to aggregate person-, stimulus- and trial-level data across studies, the benefits of data sharing for the SMID are arguably even greater than for the typical moral psychology study (especially for costly and often underpowered neuroimaging studies [38,179,226]).

#### Uses outside of moral psychology

Although the database is primarily intended for use in moral psychology, it is also worth highlighting its potential usefulness further afield. Although not primarily intended as an *affective* image set, the SMID could be used as such, given that (1) all images are normed on valence and arousal, (2) the database is more than twice the size of the largest available affective image database for which such norms are available (the NAPS [1]), and (3) given its diverse content, the database may be less vulnerable to confounds (compared for example to the GAPED [2]; see S1 Text for discussion). The SMID could therefore serve as a valuable resource for psychological and physiological investigations of emotion, and further afield, as a benchmarking or validation dataset in affective computing studies [200] (and possibly also in the fields of social computing and machine ethics [227–231]).

### Extensions

Given the large quantity of image data available on the internet, and exponential growth in Creative Commons licensed material [14,232], there is great potential to expand the image set. Extending the number of images in the dataset will become a priority as under-represented content domains (or over-used stimuli) are identified. In particular, we plan on extending the SMID to include a larger number of emotionally loaded, morally neutral stimuli, to facilitate studies that, for example, contrast emotional and moral valence.

Beyond increasing the size of the image set, there is also the prospect of collecting additional data for the images currently in the set. To this end, we are currently planning extensions to the image set including extending the set of variables for which data is available with the aim of bridging gaps between theories of moral psychology.

### Limitations

Having highlighted how the SMID could be deployed in future research, we must also acknowledge that there are applications for which the database is less well suited (e.g., contrasting first and third-person judgments [32]). Perhaps more importantly though, there are more subtle ways in which the SMID is limited, particularly regarding its representativeness of the moral domain. Whereas some of these limitations may be intrinsic to image sets in general, others may be overcome with future developments.

#### Rater representativeness

Perhaps the most obvious limitation with regards to representativeness concerns the specific population used to develop and validate the image set (i.e., AMT Workers and Australian undergraduates). Although, for the AMT sample, we recruited a politically balanced sample, there are a number of ways in which AMT samples may differ from the general population [233], which were not explicitly balanced across images. Undoubtedly, demographic factors (e.g., gender, religiosity, vegetarianism) would affect the way in which specific images are evaluated, raising interesting questions for future research.

#### Image content and representativeness

A further limitation concerning the representativeness of the SMID is that some content domains are (at least currently) less comprehensively covered than others. Taking immoral images as an example (considering, the number of images with positive uniqueness scores for each foundation, shown in Fig 5), the Fairness, Ingroup, and Authority foundations were substantially less well represented compared to Care and Purity. This is likely attributable to at least two sources: ease of portrayal and ease of retrieval.

Regarding ease of portrayal, some content domains may be more difficult to represent in the form of static images than others (much as some basic emotions are difficult to elicit with specific methods [6]). For example, a prototypical Care violation (assault) can be easily portrayed in image-form (e.g., one person punching another), whereas portraying a prototypical Ingroup violation (e.g., marital infidelity) in image-form requires communicating the presence of multiple interlinked relationships (in other words, a metarelational model [234]) such that Person A is married to Person B, who is sleeping with Person C. Thus, to the extent that specific moral content domains revolve around complex role or relationship configurations, or other abstract features that are difficult to communicate in static images (e.g., morally laden mental states, or the *simultaneous* depiction of intention, action and consequences), (1) those content domains may be represented by fewer stimuli, and (2) the stimuli that do represent those domains may do so less effectively than those representing other domains.

Regarding ease of retrieval, to the extent that suitable images for a given content domain do exist, our ability to locate them will be limited by the current ability of search engines to handle the complexity of search queries containing moral content. Such highly abstract or semantically complex queries are currently a major challenge for current image retrieval methods (e.g., [235]).

More broadly, it is worth considering the extent to which photographs are representative of everyday life. Rather than being entirely representative of everyday moral phenomena, the database is at least subtly biased towards whatever happens to attract the attention of people taking (and sharing) photographs (a phenomenon referred to as capture bias or photographer bias) [236,237]. In this particular database, there is the added potential bias introduced by any differences between people who release their photos under a Creative Commons license, and those who do not. Use of Creative Commons licensing is more common in the United States than elsewhere [14], meaning that the specific photos included in the database (and accompanying metadata) may reflect a relatively WEIRD (Western, Educated, Industrialized, Rich, Democratic) perspective [238].

An important implication of the above considerations is that, even if the SMID were representative of morally laden, permissively-licensed photographs, it would not necessarily be representative of moral behavior in general. The extent of each of these potential sources of bias, and their influence on specific uses of the database, is an important topic for future research, and an important consideration for studies using the SMID.

#### Reliance on moral foundations theory

Finally, we must consider the implications of our use of MFT in constructing and validating the SMID. Our decision to draw on MFT was pragmatic, motivated by the theory’s breadth and popularity. Although we use MFT as an organising framework, we do not argue (nor does the SMID require) that MFT provides a complete, final description of the moral universe. Although there exists research highlighting important aspects of morality potentially neglected by MFT (e.g., [122,123,239,240]), and critiquing aspects of the MFT taxonomy more generally (e.g., [90–92,241–243]), the SMID can still be used in various lines of research that are largely unrelated to MFT (e.g., [244–247]).

Most importantly, although much of the normative data refers to MFT, the *content* of the images themselves are not strictly limited to MFT-relevant content. Unlike previous stimulus sets in which materials are constrained to represent a specific theory (e.g., by excluding “ill-fitting” stimuli [26]), we made efforts to include stimuli that represent all kinds of moral content, even those that may be poorly described by MFT. As such, we anticipate the SMID to be of use to researchers working both inside and outside of MFT.

## Conclusion

Motivated by the scarcity of large, diverse, systematically validated stimulus sets available to moral psychology researchers, we developed the SMID. It is our hope that the SMID will allow researchers in the field of moral psychology to perform novel, robust and rigorous research that will ultimately make important contributions to unravelling the complexity of human moral psychology.

## Availability

The SMID images, current normative data and additional resources, can be found at https://osf.io/2rqad/. Data is currently available for all images for the following variables:

- Means, standard deviations, standard errors and rating frequencies for all five moral foundations, and valence, arousal and morality
- Uniqueness scores for all five moral foundations (as defined in the Results section for Study 2)
- An alternative Euclidean distance-based measure of uniqueness for all five moral foundations, based on [109]
- The proportion of morality ratings that were either morally good, bad, or neutral (i.e., above, below, or on the midpoint of the scale)
- Image properties including average RGB values, luminance and height-width ratio
- Image metadata including URL, title, author and license

## Supporting information

S1 Text The SMID compared to existing picture sets. (DOCX)

S2 Text Materials for image set generation. (DOCX)

S3 Text Image rating participant exclusions. (DOCX)

S4 Text Image rating instructions. (DOCX)

S5 Text Quantifying image divisiveness. (DOCX)

S1 Table OLS regressions predicting normative ratings from physical image properties. (HTML)
