# Supplementary material for: The Socio-Moral Image Database (SMID): A novel stimulus set for the study of social, moral and affective processes
Source: PLoS One. 2018 Jan 24;13(1):e0190954. doi: 10.1371/journal.pone.0190954 (PMC5783374; doi:10.1371/journal.pone.0190954)
Supplement: S1 Text — (DOCX) [file pone.0190954.s001.docx]

## S1 Text. The SMID in relation to existing affective picture sets

### Comparison of normative data

One consideration when evaluating a stimulus set concerns the size of the set, and the scope and quantity of normative data. Because no comparable moral image sets exist, we use affective image sets as a point of comparison, as presented in Table 1.

| **Table 1. Normative data quantities for the SMID and comparable affective databases** | | | | |
| --- | --- | --- | --- | --- |
| Image set | *N* images | *N* dimensions | *N* ratings / dimension | Total *N* ratings |
| SMID (Present study) | 2,941 | 8 | 35 | 820,565 |
| NAPS [1] | 1,356 | 3 | 55 | 223,740 |
| IAPS [2,3] | 1,196 | 3 | 100 | 358,800 |
| OASIS [4] | 900 | 2 | 103 | 185,400 |
| GAPED [5] | 730 | 5 | 14 | 51,100 |
| AAPS [6] | 903 | 3 | 78 | 70,434 |
| Note: SMID, Socio-Moral Image Database; NAPS Nencki Affective Picture System; IAPS, International Affective Picture System; OASIS, Open Affective Standardized Image Set; GAPED, Geneva Affective Picture Database; AAPS, Attachment Affective Picture System. In some cases, the reported rating frequencies for other image sets are approximate, based on the limited available information in the relevant citations; Subsets of the NAPS and IAPS have also been rated on additional dimensions not included here [7,8]. | | | | |

Although the number of ratings per dimension for the SMID falls within the range of existing affective image sets, the SMID does fall at the lower end of this distribution. However, this should be considered in the context of the SMID having substantially more images, and being normed on more dimensions, than existing affective image sets.

### Can the GAPED be used as a moral image set?

To our knowledge, the only systematically validated image set available that could potentially be considered a moral image set is the Geneva Affective Picture Database (GAPED [5]). The GAPED contains 730 images divided into six *a priori* categories: four separate groups of negatively valenced images containing (1) spiders, (2) snakes, (3) human rights violations, and (4) animal mistreatment, along with heterogeneous collections of (5) positive, and (6) neutral images.

Although not developed specifically for moral psychology research, the inclusion of two morally negative categories, human rights violations and animal mistreatment, suggests that it could be used as such. However, the GAPED is severely limited as a tool for moral psychology research, given that (1) its moral content ratings (restricted to the two dimensions of moral/ethical and legal acceptability) are only available for the two “moral” image categories and not the four non-moral categories (meaning that only 229 of the 730 images have norms for moral content), and (2), there is severe confounding with non-moral image features. For example, 101 out of 105 (i.e., 96%) of human rights violation images contain people whereas only five of 89 neutral images (i.e., 6%), and none of the 291 negative “non-moral” images (i.e., snake and spider images) do.

In other words, if one were to use the GAPED in a study contrasting immoral images with either neutral or non-moral negative images, that contrast will almost inevitably confounded with a contrast between images containing humans or (non-snake and non-spider) animals on the one hand, and images containing either spiders, snakes or no humans or animals at all. As such, the GAPED is likely of limited use as a tool for moral psychology research.

## References

1. Marchewka A, Zurawski Ł, Jednoróg K, Grabowska A. The Nencki Affective Picture System (NAPS): introduction to a novel, standardized, wide-range, high-quality, realistic picture database. Behav Res Methods. 2014;46: 596–610. doi:10.3758/s13428-013-0379-1

2. Lang PJ, Bradley MM, Cuthbert BN. International affective picture system (IAPS): Affective ratings of pictures and instruction manual. Technical Report A-8. Gainesville, FL; 2008.

3. Bradley MM, Lang PJ. The International Affective Picture System (IAPS) in the study of emotion and attention. In: Coan JA, Allen JJB, editors. Handbook of Emotion Elicitation and Assessement. New York: Oxford University Press; 2007. pp. 29–46.

4. Kurdi B, Lozano S, Banaji MR. Introducing the Open Affective Standardized Image Set (OASIS). Behav Res Methods. 2016; doi:10.3758/s13428-016-0715-3

5. Dan-Glauser ES, Scherer KR. The Geneva affective picture database (GAPED): A new 730-picture database focusing on valence and normative significance. Behav Res Methods. 2011;43: 468–77. doi:10.3758/s13428-011-0064-1

6. Liu Y, Chen X, Zhai J, Tang Q, Hu J. Development of the attachment affective picture system. Soc Behav Pers. 2016;44: 1565–1574. doi:10.2224/sbp.2016.44.9.1565

7. Libkuman TM, Otani H, Kern R, Viger SG, Novak N. Multidimensional normative ratings for the International Affective Picture System. Behav Res Methods. 2007;39: 326–334. doi:10.3758/BF03193164

8. Riegel M, Żurawski Ł, Wierzba M, Moslehi A, Klocek Ł, Horvat M, et al. Characterization of the Nencki Affective Picture System by discrete emotional categories (NAPS BE). Behav Res Methods. 2016;48: 600–612. doi:10.3758/s13428-015-0620-1
