# Supplementary material for: The Socio-Moral Image Database (SMID): A novel stimulus set for the study of social, moral and affective processes
Source: PLoS One. 2018 Jan 24;13(1):e0190954. doi: 10.1371/journal.pone.0190954 (PMC5783374; doi:10.1371/journal.pone.0190954)
Supplement: S2 Text — (DOCX) [file pone.0190954.s002.docx]

## S2 Text. Materials for image set generation.

### Image Search Instructions

Before commencing the image search task, participants were asked to read the following instructions:

We are developing a set of images that relate to particular concepts which we can use in our research. To assist in this process, what we would like you to do is perform a web-search for **10** photographic images that you think would make most people think of each the following concepts:

- **10** images representing **[CONCEPT_1]**
- **10** images representing **[CONCEPT_2]**

This means that you will be searching for a total of **20** images.

*What sort of images should I search for?*

The images you search for need to match a specific set of criteria, namely they:

- Must be **photographic** images (i.e. of actual scenes, events, etc.) as opposed to drawings or other kinds of images
  - When using Google Images, after beginning your search, you can filter images by clicking on "*Search tools*," then clicking on "*Type*," and selecting "*Photo*" to filter out images that don't meet the criteria (the filter isn't perfect, but it may help)
- Must **not** contain obscene or offensive content (e.g. pornographic content or graphic portrayals of violence)
- Must have a minimum resolution of 640*480
  - Again, when using Google Images, you can filter images by clicking on "*Search tools*," then clicking on "*Size*," and selecting "*Larger than...*", and selecting "*640x480*."
- Must be in one of the following file formats:
  - JPEG
  - BMP
  - PNG
  - GIF
- Must **not** contain watermarks
- Must **not** contain as a central feature any of the following:
  - Commercial logos
  - Famous places
  - Famous people
  - Text
- Must be images which can be accessed by anyone by simply entering a URL for the image (i.e. the images aren't stored on a website that can only be accessed, for example, by registered users)
- Must be licensed under Creative Commons or GNU Free Documentation licenses (GFDL)
  - When using Google Images, after entering your initial search terms and bringing up results, you can filter images by clicking on "*Search tools*," then clicking on "*Usage rights*," and selecting "*Labeled for reuse*" (for further information, see [here](http://www.unimelb.edu.au/copyright/information/guides/googleimagesblue.pdf))

*Where should I search?*

For this task, we would like you to search either Flickr or the Wikimedia commons. You may find it easiest to use resources such as Google Images to do this. To make the task as easy as possible, this link [here](https://www.google.com.au/search?as_st=y&tbm=isch&hl=en&as_q=circle&as_epq=&as_oq=&as_eq=&cr=&as_sitesearch=flickr.com/&safe=images&tbs=isz:lt,islt:vga,itp:photo,sur:fc) (for Flickr) or [here](https://www.google.com.au/search?q=circle+site:commons.wikimedia.org/&safe=images&as_st=y&hl=en&tbs=sur:fc,isz:lt,islt:vga,itp:photo&tbm=isch&source=lnt&sa=X&ei=SEOZU6vUFYnzkQXVg4GoAQ&ved=0CBQQpwU&dpr=1&biw=1440&bih=769) (for Wikimedia) will direct you to an example Google Image search for the term "circle" with the filters described above applied. All you need to do is change the search term to match whatever kind of image you want to find. As stated above, the images must be publicly available, not offensive, and accessible using the URL which you enter in the space provided.

*How do I return the image I have found?*

For each image you find, there will be a text box in which you can paste the URL for the image. These URLs can usually be retrieved (for example, on a Windows PC using Firefox) by right-clicking on the image and selecting "*Copy Link Location*." These URLs will then be used by us to collect the images.

Please note that **the URL must end with the file extension for the image** (e.g. www.example.com/image**.jpg**). URLs formatted in different ways will **not** work.

Please note that these instructions **will still be available** after you leave this page. Thank you for your participation.

### Concept Generation Instructions

Before beginning the image search, participants were asked to complete the following concept generation task to increase image diversity (i.e., to prevent participants from simply using their concept label as the sole search term and returning the first results from that search).

Before beginning the main task, we would like you to think briefly about the things that you associate with the concepts of [CONCEPT_1] and [CONCEPT_2] that are most important to you.  Using each of the fields below, we would like you to briefly list some of the things that come to mind when you think about these concepts.  These will be used later as prompts for image searches that you will perform.

The things you list might include events, objects, people, places, or anything else, and these could be positive, negative, or neutral.

### Concept Search Instructions

When completing the image search task, the following instructions were displayed:

Following the instructions you read earlier (repeated below, if you'd like to re-read them), please find an image that you believe would make most people think of [CURRENT CONCEPT].

To help with your search, please make use of the list of events, objects, etc. that you generated at the beginning of the task. (Please note that you don't strictly have to use these search terms - these are just to help you think about what sort of images might be used to portray or communicate [CURRENT CONCEPT].)
