# Supplementary material for: The Socio-Moral Image Database (SMID): A novel stimulus set for the study of social, moral and affective processes
Source: PLoS One. 2018 Jan 24;13(1):e0190954. doi: 10.1371/journal.pone.0190954 (PMC5783374; doi:10.1371/journal.pone.0190954)
Supplement: S3 Text — (DOCX) [file pone.0190954.s003.docx]

## S3 Text. Image rating participant exclusions.

Our initial sample comprised 3,293 datasets. From this, we excluded participants from all analyses (including the computing of image norms) if they either provided incomplete data (*N* = 18 AMT participants; 19 undergraduates), failed multiple catch trials (*N* = 153 AMT participants; 190 undergraduates; procedure described below), provided response times of less than 150 MS for at least five trials (*N* = 34 AMT participants; 13 undergraduates),^[[1]](#footnote-1)^ or spent less than five seconds reading the descriptions of each five moral foundation dimensions (*N* = 98 AMT participants; 52 undergraduates; described below). In total, this resulted in the exclusion of 577 participants.

In addition, because we did not restrict AMT participants from completing the task multiple times, we identified 596 datasets that belonged to participants who had already completed the task at least once (on average, AMT participants completed the task 1.17 times). Only one AMT participant completed the exact same batch twice. Their data from the second completion was excluded. Because all other participants were always assigned to rate different batches of images, we simply retained their image rating data by merging it with any previous data they had provided.

## Catch Trials

To determine whether participants were paying attention, we embedded five catch trials in the image rating task. These trials entailed the presentation of images (such as a blank billboard) where text had been inserted, instructing participants to press a specific button to demonstrate that they were paying attention. The five catch trials were randomly distributed amongst separate blocks (no more than one trial per block, never during the first block) and presented on a randomly selected trial. While we had originally intended to exclude participants failing two or more catch trials, one catch stimulus accounted for a disproportionate number of errors (i.e., was substantially more difficult; see Fig 1). To avoid unnecessarily discarding useable data, we opted to only exclude participants based on responses to the other four stimuli.

**Fig 1. Proportion of correct responses across catch trial images.**

## Instruction Reading Times

At the end of the study participants were asked to estimate the number of times they had completed the Moral Foundations Questionnaire [1]. Because many AMT participants reported a high degree of familiarity with the MFQ (169 participants reported at least 10 MFQ completions), we decided to exempt AMT participants who reported having completed the MFQ at least 10 times from the read time exclusion. This resulted in the inclusion of three participants who would have otherwise been excluded.

## References

1. Graham J, Nosek BA, Haidt J, Iyer R, Koleva SP, Ditto PH. Mapping the moral domain. J Pers Soc Psychol. 2011;101: 366–85. doi:10.1037/a0021847

1. In addition, among remaining participants, 244 individual trials with RTs < 150 MS were deleted. [↑](#footnote-ref-1)
