# Supplementary material for: The Socio-Moral Image Database (SMID): A novel stimulus set for the study of social, moral and affective processes
Source: PLoS One. 2018 Jan 24;13(1):e0190954. doi: 10.1371/journal.pone.0190954 (PMC5783374; doi:10.1371/journal.pone.0190954)
Supplement: S4 Text — (DOCX) [file pone.0190954.s004.docx]

# S4 Text. Image rating instructions.

Before rating each dimension, participants were instructed to read the corresponding detailed description provided below.

### Care/Harm

#### What is it?

- This is a category of moral values that relates to harming or caring for others
- Actions are deemed wrong because they concern harming others (physically, psychologically, and/or emotionally)
- Actions are deemed right because they concern helping, caring for, protecting, or showing compassion towards others
- Central concepts include care, compassion, empathy, help, support; harm, cruelty, emotional suffering, weakness/vulnerability

#### How to give your responses

- Note that you are not rating whether something is a positive or negative instance of this value - clear examples of HARM and CARE should both score highly
- Also note that the image need not represent all aspects of the category to get a high rating - it could just represent one aspect very clearly
- If you think the image clearly represents some aspect of this moral value (moral or immoral), respond with a 5
- If you think the image is completely irrelevant to this moral value, respond with a 1
- Use the intermediate options to indicate images that elicit a response somewhere in between

### Fairness/Cheating

#### What is it?

- This is a category of moral values that relates to justice, fairness, rights, and reciprocity
- Actions are deemed wrong because they cause injustice, unfairness, or lack of reciprocity, and/or violate individual rights
- Actions are deemed right because they uphold justice, fairness, or reciprocity, and/or uphold individual rights
- Central concepts include fairness, reciprocity, justice, rights, equality, equity, proportionality, honesty; discrimination, cheating, bias, lying

#### How to give your responses

- Note that you are not rating whether something is a positive or negative instance of this value - clear examples of FAIRNESS and CHEATING should both score highly
- Also note that the image need not represent all aspects of the category to get a high rating - it could just represent one aspect very clearly
- If you think the image clearly represents some aspect of this moral value (moral or immoral), respond with a 5
- If you think the image is completely irrelevant to this moral value, respond with a 1
- Use the intermediate options to indicate images that elicit a response somewhere in between

### Loyalty/Betrayal

#### What is it?

- This is a category of moral values that relates to the obligations of group membership such as loyal duty and commitment to others (often to members of one's group, e.g., family, country, friendship group, team, or some other social group)
- Actions are deemed wrong because some duty or commitment to others has been neglected
- Actions are deemed right because they uphold obligations, duties or loyalties to others
- Central concepts include loyalty, unity, solidarity, alliance, ingroup, mateship, teamwork; betrayal, disloyalty, traitors

#### How to give your responses

- Note that you are not rating whether something is a positive or negative instance of this value - clear examples of LOYALTY and BETRAYAL should both score highly
- Also note that the image need not represent all aspects of the category to get a high rating - it could just represent one aspect very clearly
- If you think the image clearly represents some aspect of this moral value (moral or immoral), respond with a 5
- If you think the image is completely irrelevant to this moral value, respond with a 1
- Use the intermediate options to indicate images that elicit a response somewhere in between

### Respect/Subversion

#### What is it?

- This is a category of moral values that relates to the obligations of hierarchical relationships such as showing respect to others, and/or showing proper deference/duty to a superior
- Actions are deemed wrong because somebody is being disrespectful, and/or is undermining the leadership of the superior in that particular relationship or situation
- Actions are deemed right because somebody shows proper respect to others, and/or is showing proper deference to the superior in the particular relationship or situation
- Central concepts include respect, esteem, honor, roles, order, leadership, deference; disrespect, disobedience, insubordination, subversion

*NOTE:* 'Superior' can relate to the relationship, e.g., a boss is an employee's superior, a parent is a child's superior. 'Superior' can sometimes also depend on the situation. E.g., if one person is giving advice/direction/instruction to another, he/she is the 'superior' in that particular situation/task.

#### How to give your responses

- Note that you are not rating whether something is a positive or negative instance of this value - clear examples of RESPECT and SUBVERSION should both score highly
- Also note that the image need not represent all aspects of the category to get a high rating - it could just represent one aspect very clearly
- If you think the image clearly represents some aspect of this moral value (moral or immoral), respond with a 5
- If you think the image is completely irrelevant to this moral value, respond with a 1
- Use the intermediate options to indicate images that elicit a response somewhere in between

### Sanctity/Degradation

#### What is it?

- This category of moral values emphasizes the purity and sanctity of human beings
- Actions are deemed wrong because they threaten to defile the sanctity and decency of the human body, soul, or spirit
- Actions are deemed right because they maintain purity and sanctity and prevent physical or spiritual contagion
- Central concepts include purity, sanctity, decency, piety, sacredness, cleanliness, wholesomeness; unnaturalness, depravity, sin, lewdness, defilement

#### How to give your responses

- Note that you are not rating whether something is a positive or negative instance of this value - clear examples of SANCTITY and DEGRADATION should both score highly
- Also note that the image need not represent all aspects of the category to get a high rating - it could just represent one aspect very clearly
- If you think the image clearly represents some aspect of this moral value (moral or immoral), respond with a 5
- If you think the image is completely irrelevant to this moral value, respond with a 1
- Use the intermediate options to indicate images that elicit a response somewhere in between

### Morality/Immorality

#### What is it?

- This concept simply relates to how moral or immoral you find the content of the image

#### How to give your responses

- Note that the image need not portray an action to get a high or low rating - it could instead be a symbol or some other representation of morality or immorality
- You should respond to extremely immoral images with a 1, and extremely moral images with a 5
- Moderately immoral and moral images should get responses of 2 and 4
- If the image seems completely morally neutral, respond with a 3

### Pleasantness

#### What is it?

- This concept simply relates to how pleasant you find the content of the image

#### How to give your responses

- You should respond to extremely unpleasant images with a 1, and extremely pleasant images with a 5
- Moderately unpleasant and pleasant images should get responses of 2 and 4
- If the image seems completely neutral, respond with a 3

### Excitement/Arousal

#### What is it?

- This concept simply relates to how exciting or arousing you find the content of the image
- Note that it need not be exciting in the positive, enjoyable sense, but rather that it simply elicits a strong response
- Examples of this include the feeling most people would experience in response to rollercoaster rides, winning the lottery or seeing a snake or horror movie

#### How to give your responses

- If you find the image doesn't elicit any kind of strong response, respond with a 1
- If the image elicits and extremely strong response, respond with a 5
- Use the intermediate options to indicate images that elicit a response somewhere in between
