# Supplementary material for: The Socio-Moral Image Database (SMID): A novel stimulus set for the study of social, moral and affective processes
Source: PLoS One. 2018 Jan 24;13(1):e0190954. doi: 10.1371/journal.pone.0190954 (PMC5783374; doi:10.1371/journal.pone.0190954)
Supplement: S5 Text — (DOCX) [file pone.0190954.s005.docx]

## S5 Text. Quantifying image divisiveness

As mentioned in the main text, one interesting source of variability in the image rating data is divisiveness: disagreement regarding some evaluative aspect of an image, absent any ambiguity or disagreement about its content (for example, an image portraying a same-sex wedding). Given the available data, one method of identifying divisive images is to examine the extent to which variance in ratings on a given dimension are explained by some grouping variable: an image is likely to be divisive to the extent that (1) judgements of an image within a group are in agreement (e.g., within political conservatives), while (2) judgements across groups are in disagreement (e.g., when comparing liberals with conservatives). Defined as such, divisiveness can be measured in a conventional ANOVA (or regression) design. Thus, to quantify divisiveness, we conducted separate one-way ANOVAs on each of the 2,941 images, on each of the eight dimensions, on the following four grouping variables: political orientation (separately: general conservatism, social conservatism and economic conservatism), and gender.

To measure divisiveness, we use the effect size from each ANOVA, representing the proportion of variance explained by the grouping variable. Images that are divisive with respect to a grouping variable (e.g., an image of a same-sex marriage, comparing responses from liberals and conservatives) would be expected to have larger effect sizes than images that are not divisive. Because the number of observations varies across images, we use ω^2^ as our effect size metric rather than the more familiar *η*^2^, because of *η*^2^’s substantial upward bias in small samples [1,2] (and the substantial reduction in bias that can be achieved by using ω^2^).^[[1]](#footnote-1)^ This is particularly important in our case where images vary widely in the number of ratings they receive.

Because of the enormous number of comparisons performed (2,941 images × 8 dimensions × 4 grouping variables = 94,112 ANOVAs), we refrain from performing significance tests. Instead, the analyses presented in this section are merely intended to (1) demonstrate one possible approach to measuring divisiveness, and (2) provide a starting point for future research interested in identifying and using divisive images. The ω^2^ values produced from these analyses can be obtained from the SMID Open Science Framework repository (<https://osf.io/2rqad/>).

## Results

To demonstrate our approach, we limit our presentation of analyses of divisiveness to two dimension-group combinations: (1) morality ratings, segregated by political orientation (based on dividing responses to a 9-point general political orientation question into three groups: Liberal [1,2,3,4], Moderate [5], and Conservative [6,7,8,9]), and (2) valence ratings, segregated by gender. Additional results can be obtained from <https://osf.io/2rqad/>. Density plots depicting distributions of ω^2^ for these two analyses across the entire image set, are presented in Fig 1.

Fig 1. Density plots of image divisiveness.

Perhaps the most salient feature of Fig 1 is that most ω^2^ values for both analyses are at or below zero, indicative of no systematic differences across groups in the overwhelming majority of cases: for variance in morality ratings explained by political orientation, only 25% of images had ω^2^ values greater than .01 (and for valence ratings explained by gender, such was the case for only 10% of images). The positive tails of the distributions, however, suggest the presence of a small number of images for both dimension-group combinations where systematic variance may be present. To allow a closer inspection, we extracted the five most divisive images for both analyses. These images are presented along with summary statistics in Fig 2 and Fig 3 below.

Fig 2. Top five divisive images for moral ratings disaggregated by political orientation. Left column contains each of the top five images and their SMID identifier. Right column contains group means for moral ratings for liberals (L), moderates (M) and conservatives (C), with ω^2^ inset. Error bars represent ±2SE.

Fig 3. Top five divisive images for valence ratings disaggregated by gender. Left column contains each of the top five images and their SMID identifier. Right column contains group means for gender for females (F) and males (M), with ω^2^ inset. Error bars represent ±2SE.

## Limitations

Note that the simple analyses presented here do not account for the potential confounding of grouping variables. For example, younger participants – especially those in the undergraduate sample – may be more politically liberal. If ratings of some images varied systematically with age, then the confounding of age with political orientation would likely result in the identification of images that are *apparently* divisive with respect to political orientation, but in reality, are not (but are instead divisive with respect to age). To address this issue, the analyses presented here could straightforwardly be extended to accommodate multiple grouping variables, however we leave this for future research.

## References

1. Okada K. Is omega squared less biased? A comparison of three major effect size indices in one-way ANOVA. Behaviormetrika. 2013;40: 129–147. doi:10.2333/bhmk.40.129

2. Lakens D. Calculating and reporting effect sizes to facilitate cumulative science: a practical primer for t-tests and ANOVAs. Front Psychol. 2013;4: 1–12. doi:10.3389/fpsyg.2013.00863

1. Note that, because ω^2^ attempts to correct for sampling error, some effect sizes will be negative (but in the presence of a null effect, we would expect these negative values to approach zero as more data is collected). Given the impossibility of the proportion of explained variance actually being below zero, negative values can simply be interpreted as suggesting a null effect (i.e., ω^2^ = 0). [↑](#footnote-ref-1)
