# Supplementary material for: The Socio-Moral Image Database (SMID): A novel stimulus set for the study of social, moral and affective processes
Source: PLoS One. 2018 Jan 24;13(1):e0190954. doi: 10.1371/journal.pone.0190954 (PMC5783374; doi:10.1371/journal.pone.0190954)
Supplement: S1 Table — (HTML) [file pone.0190954.s006.html]

OLS Regressions predicting normative ratings from physical image properties

|  | **Care** | **Fairness** | **Ingroup** | **Authority** | **Purity** | **Moral** | **Valence** | **Arousal** |
| Intercept | **2.97**\* | **2.17**\* | **2.57**\* | **2.36**\* | **2.48**\* | **3.05**\* | **3.13**\* | **2.72**\* |
|  | [2.81; 3.12] | [2.06; 2.29] | [2.42; 2.72] | [2.23; 2.50] | [2.36; 2.60] | [2.92; 3.18] | [2.97; 3.29] | [2.61; 2.84] |
| Red | -0.57 | **-0.54**\* | -0.61 | -0.33 | -0.22 | 0.01 | 0.05 | -0.13 |
|  | [-1.21; 0.07] | [-1.02; -0.05] | [-1.23; 0.02] | [-0.89; 0.22] | [-0.73; 0.29] | [-0.55; 0.56] | [-0.62; 0.72] | [-0.62; 0.36] |
| Green | -0.37 | -0.20 | -0.20 | -0.09 | -0.31 | 0.02 | 0.13 | -0.21 |
|  | [-1.05; 0.32] | [-0.72; 0.32] | [-0.87; 0.47] | [-0.69; 0.51] | [-0.86; 0.24] | [-0.57; 0.61] | [-0.58; 0.85] | [-0.73; 0.31] |
| Blue | 0.95 | 0.76 | 0.89 | 0.45 | 0.56 | -0.04 | -0.26 | 0.28 |
|  | [-0.05; 1.96] | [-0.01; 1.52] | [-0.09; 1.87] | [-0.43; 1.32] | [-0.25; 1.37] | [-0.90; 0.83] | [-1.32; 0.79] | [-0.49; 1.04] |
| Contrast | **0.67**\* | **1.38**\* | **1.32**\* | **1.98**\* | **1.41**\* | 0.19 | -0.34 | **0.52**\* |
|  | [0.02; 1.32] | [0.88; 1.87] | [0.69; 1.96] | [1.41; 2.54] | [0.89; 1.93] | [-0.37; 0.75] | [-1.02; 0.34] | [0.03; 1.01] |
| R2 | 0.00 | 0.01 | 0.01 | 0.02 | 0.01 | 0.00 | 0.00 | 0.00 |
| Adj. R2 | 0.00 | 0.01 | 0.01 | 0.02 | 0.01 | -0.00 | 0.00 | 0.00 |
| Num. obs. | 2941 | 2941 | 2941 | 2941 | 2941 | 2941 | 2941 | 2941 |
| RMSE | 0.94 | 0.71 | 0.92 | 0.81 | 0.75 | 0.81 | 0.98 | 0.71 |
| \* 0 outside the confidence interval. Parameter estimates are unstandardized. | | | | | | | | | |
